# Response: Commentary: Evaluating Sensory Integration/Sensory Processing Treatment: Issues and Analysis

**DOI:** 10.3389/fnint.2022.874320

**Published:** 2022-06-07

**Authors:** Sarah A. Schoen, Roseann C. Schaaf, Zoe Mailloux, Anita Bundy, Shelly Lane, Teresa A. May-Benson, L. Diane Parham, Susanne Smith Roley

**Affiliations:** ^1^STAR Institute for Sensory Processing, Centennial, CO, United States; ^2^Rocky Mountain University of Health Professions, Provo, UT, United States; ^3^Jefferson Autism Center of Excellence, Thomas Jefferson University School of Rehabilitation Sciences and Department of Occupational Therapy, Philadelphia, PA, United States; ^4^Department of Occupational Therapy, Colorado State University College of Health and Human Sciences, Fort Collins, CO, United States; ^5^TMB Education, LLC, Norristown, PA, United States; ^6^Institute of Occupational Therapy Education, Widener University, Chester, PA, United States; ^7^University of New Mexico, Albuquerque, NM, United States; ^8^Collaborative for Leadership in Ayres Sensory Integration, Aliso Viejo, CA, United States

**Keywords:** sensory integration, sensory processing, evidence-based intervention, treatment effect analysis, treatment outcome

## Introduction

As experts in the field of sensory integration, we were eager to read Camarata et al.'s paper Evaluating Sensory Integration/Sensory Processing Treatment: Issues and Analysis. Accurate representation of sensory integration and its effectiveness are essential for consumers and researchers, as this will provide a clear and useful path forward. Unfortunately, this manuscript misrepresents current evidence, which will add confusion, rather than clarity to the science. The authors have inaccurately characterized the intervention components, employed language not used in the field, and proposed an inappropriate framework for systematic testing. They advocate for fair trials that are inconsistent with the theory of change presented and include outcomes irrelevant to the intervention. Below, we highlight some of the main areas in which Camarata et al.'s review falls short of an accurate analysis and why their suggested next steps neglect to build on the existing evidence base.

## Evaluation of the Evidence

Ayres Sensory Integration® (ASI) is a multifaceted, complex theory and intervention approach. The core elements of ASI are active engagement of the child in individually tailored sensory motor activities, contextualized in play, at the “just right” challenge in a therapeutic alliance. The desired outcomes of ASI are improved processing and integration of sensation to enable adaptive responses that support meaningful engagement in everyday life activities.

The term Ayres Sensory Integration® was registered specifically to distinguish it from other sensory-based protocols/ procedures or intervention (SBIs) (Smith Roley et al., 2007). Unfortunately, Camarata and colleagues inappropriately combined evidence for ASI® with that of other sensory-based interventions such as sensory diets, and single domain sensory protocols. These sensory-based interventions are restricted protocols conducted by caregivers or school personnel designed to target specific sensory symptoms (Case-Smith et al., 2015). While they utilize sensory input, the application of these interventions is inconsistent with the core elements of ASI.

Importantly, the ASI® Fidelity Measure (Parham et al., 2007, 2011; May-Benson et al., 2014) details the core elements of ASI and explicates the essential features of this approach. This validated tool guides clinicians and researchers to distinguish ASI from sensory-based protocols. When evaluating the evidence from individual studies or systematic reviews, the ASI Fidelity Measure (Case-Smith et al., 2015; Bundy and Lane, 2020) should be used to distinguish studies of ASI intervention from other sensory interventions. Failure to use this tool to distinguish studies is a major flaw in Camarata et al.'s paper. In short, their finding of “mixed results” for sensory integration interventions does not reflect the current state of knowledge/evidence about ASI (Schaaf et al., 2018; Schoen et al., 2019; Steinbrenner et al., 2020; Hume et al., 2021). Rather, current evidence, including randomized controlled trials and single subject case studies, supports the effectiveness of ASI (Miller et al., 2007; Pfeiffer et al., 2011; Schaaf et al., 2014; Kashefimehr et al., 2018; Schoen et al., 2018, 2019; Andelin et al., 2021; Omairi et al., 2022, 2022).

## Theory of Change

In their discussion of *theory of change* as it applies to ASI, Camarata and colleagues again inaccurately represented the literature. Their models presented in Figures 1 and 3 suggest a linear progression from sensory to motor to social to behavior and from tactile stimulation to improved play to increased social learning. This is a vastly over-simplified depiction of dysfunction and intervention and an inaccurate portrayal of the processes described in ASI. ASI theory considers the complex, dynamic, multidirectional nature of sensory reception, integration, analysis and output (Schaaf and Mailloux, 2015 Chapter 1; Bundy and Lane, 2020) and is based on developmental theory of sensorimotor functions and principles of neuroplasticity.

The theory of change underpinning ASI considers the ability of the central nervous system to change, the role of active participation, interdependency of the body-centered senses and the integration of sensation from movement and the environment, all of which support planning and organizing of behavior. Engagement in a trusting/safe relationship (i.e., a therapeutic alliance) is critical to the theory of change in ASI (Lane and Schaaf, 2010; Reynolds et al., 2010; Kilroy et al., 2019; Lane et al., 2019).

In short, ASI involves active engagement of child and therapist in a therapeutic relationship. The interactive social components of this alliance require collaboration and communication that are essential to the intervention. Separating these therapeutic components from the sensory-motor activities, as Camarata et al. suggested, is not possible in the ASI approach. While, it *is* possible to deliver sensory-based activities without verbal/nonverbal transactions, this type of modification is inconsistent with the active ingredients of ASI. Nonetheless, a randomized controlled trial comparing conversational recasting to ASI *might be* a viable means of examining differences in outcomes between these differing approaches.

## Outcomes: Conducting Fair Trials

ASI is a well-established, complex, sensory motor intervention that is individually tailored to the needs of each child and family (Schaaf and Mailloux, 2015). The content of intervention is based on a comprehensive assessment of sensory integration and the outcomes are improved participation in daily life activities and tasks. The ultimate goal is improved quality of life of children and their families characterized by improved function and participation in daily activities, roles, and routines (Schaaf et al., 2011, 2015; Schaaf and Mailloux, 2015; Ismael et al., 2018; Schaaf and Mailloux in Bundy and Lane, 2020). A professional, most commonly an occupational therapist, with advanced training in the theory and approach is the interventionist (Steinbrenner et al., 2020).

Implementation of ASI considers both proximal and distal outcomes. Distal outcomes are skills, abilities and behaviors expected to change as a result of intervention often without being directly targeted, while proximal outcomes are the underlying sensory-motor factors hypothesized to impact distal outcomes (Melnyk and Morrison-Beedy, 2012; Schaaf and Mailloux, 2015). With its focus on function, distal outcomes of ASI reflect improvements in participation/functioning in daily life (e.g., completing family chores or independently performing a bath-time or bedtime routine). Proximal markers reflect change in sensory and motor factors hypothesized to underlie participation/functional challenges (e.g., improved posture, balance, sensory perception and praxis; Schaaf, 2015). The link between proximal and distal outcomes is key in ASI. Camarata and colleagues missed this important point when discussing outcome measures. They proposed multisensory integration (MSI) as a measure but failed to link this proximal outcomes to distal, participation-based outcomes. While changes in MSI *might* be an appropriate proximal marker for some children, the MSI measure of auditory-visual integration is not appropriate. Tactile, vestibular and proprioceptive sensory perception and integration, rather than visual and auditory perception and integration, have always been the focus of ASI (Ayres, 1972). A *fair* trial would include the primary sensory domains addressed within ASI, with both proximal (sensory-motor or MSI) and distal (function and participation-based) outcomes.

## Discussion

ASI is an evidence-based intervention comprising elements specified in the fidelity measure and effectiveness determined through measurable distal outcomes (Steinbrenner et al., 2020). We agree with the call for more research into ASI and assert there is a solid foundation of evidence upon which to build. We further suggest that the research community spend less time deconstructing existing evidence and more time conducting new research that builds on ASI's existing foundation.

As with any intervention, continued research is needed to identify the mechanisms of action, the markers of change and the populations for which the intervention is effective. However, this work must utilize appropriate interpretations of existing literature, particularly the rigorous studies published in the last decade. The literature is fraught with inaccuracies, discrepancies in terminology and misrepresentations of sensory integration as a therapeutic practice. A sophisticated analysis is needed to assure that conclusions are accurate and scientifically sound. We contend that the Camarata et al. review failed to provide an accurate representation of ASI; failed to appreciate the complex nature of the theory and intervention approach; and proposed a strategy for evaluating change that is not consistent with the theoretical tenants of ASI. Our response to this article is provided in the spirit of clarification. Our intent is to challenge scientists and clinicians to be thoughtful and systematic when designing future studies and evaluating interventions addressing sensory differences that impact function and participation.

## Author Contributions

SS, RS, and ZM provided substantial contributions to the conception or design of the work, or the acquisition, and analysis or interpretation of data for the work. AB, SL, TM-B, LP, and SR assisted in drafting the work or revising it critically for important intellectual content as well as providing approval for publication of the content. All authors agree to be accountable for all aspects of the work in ensuring that questions related to the accuracy or integrity of any part of the work are appropriately investigated and resolved.

## Conflict of Interest

The authors declare the following Conflicts of Interest (Bottema-Beutel et al., 2021): SS was involved in the development of an intervention based on the principles of ASI. She is employed by a clinic that offers ASI as a direct service and which trains others to use the intervention. She does not receive royalties for the sale of materials or personal fees for paid workshops. RS received grant funding to support research on ASI. She trains individuals in ASI intervention and receives royalties for the sale of materials she created. She will seek funding from Thomas Jefferson University to support the publication of this manuscript. ZM receives monetary compensation from her teaching for both the Collaborative for Leadership in ASI and for Thomas Jefferson University both of which are entities, which provide training in ASI intervention. She also receives royalties for the sale of materials she created. AB attests that she receives monetary compensation from the sale of ASI materials she created. SL attests that she receives monetary compensation from the sale of ASI materials she created. She provides training on the Neuroscience Basis for ASI and receives honoraria. She will seek funding from an internal research fund at Colorado State University to pay for this article's publication fee. TM-B developed two interventions based on ASI, Safe Place- An ASI based trauma informed intervention and Sensory Bridges to Social Competency- an ASI based sensory motor social group intervention. The author owns and is employed by company TMB Education, LLC., that provides ASI. She also trains others to use the intervention and receives speaker fees for the delivery of this content. LP is a co-author of the Sensory Processing Measure (SPM and SPM-2) assessments published by Western Psychological Services. She receives monetary royalties for the sale of these products, and occasionally receives compensation for teaching workshops on how to use the SPM assessments. SR is affiliated with an OT clinic that provides ASI but is not employed by the entity. She receives compensation for training others to use the intervention as well as royalties from publications and speaker fees through CLASI.

## Publisher's Note

All claims expressed in this article are solely those of the authors and do not necessarily represent those of their affiliated organizations, or those of the publisher, the editors and the reviewers. Any product that may be evaluated in this article, or claim that may be made by its manufacturer, is not guaranteed or endorsed by the publisher.
